# The Effect of Ostracism on Adults’ Materialism: The Roles of Security and Self-Construal

**DOI:** 10.3389/fpsyg.2022.796924

**Published:** 2022-04-18

**Authors:** Jiaxi Feng, Yichen Wang, Zhiyu Ji, Denghao Zhang

**Affiliations:** Department of Psychology, Renmin University of China, Beijing, China

**Keywords:** ostracism, materialism, security, self-construal, adults

## Abstract

With consumer culture becoming more prominent, the value of materialism is growing rapidly. This study explored the formation of materialism in adults, based on the temporal need–threat model of ostracism and the theory of materialistic values. Specifically, this study examined the link between ostracism and materialism from the perspective of security and the moderating role of self-construal in this process. A sample of 1,272 Chinese adults (*M*_age_ = 35.90 ± 11.59, 47.2% male) was recruited to complete the Ostracism Experiences Scale, the Material Values Scale, the Security Questionnaire, and the Self-Construal Scale. The results showed that (1) ostracism positively predicted materialism in Chinese adults; (2) security partially mediated the relationship between ostracism and materialism; (3) and self-construal moderated this mediation model. The moderating effect of self-construal on the relationship between ostracism and security was significant. Specifically, the direct effect of ostracism on security was much stronger for adults with interdependent self-construal than for those with independent self-construal. However, self-construal had no significant moderating effect on the direct effect of ostracism on materialism. These findings suggest that ostracism may affect materialism by damaging adults’ feelings of security, and independent self-construal can buffer the damage of ostracism on security.

## Introduction

With consumer culture becoming more prominent, a standard of success is often measured by how many material possessions one has (Duh, 2015). Materialism emphasizes the importance of material possessions (Richins, 2004). However, one notably consistent finding is that excessive pursuit of material possessions is detrimental to the self and society. Materialists are less happy and have more depressive symptoms (Muñiz-Velázquez et al., 2017). They care less about public interest and environmental issues (Li et al., 2020), and even show more antisocial behavior (Kasser, 2016). Therefore, to prevent and reduce materialism, the mechanism causing materialism needs to be understood.

Previous studies on the causes of materialism focused on mental factors (such as security and self-esteem, Kasser et al., 2004; Zhang and Hawk, 2019), social and cultural factors (such as social class and media, Duh, 2015), and interpersonal environment factors (such as parental conflict and peer relationship; Jiang et al., 2015; Ching and Wu, 2018). Although previous studies have investigated the interpersonal environment factors, the focus was on children and adolescents, limiting the investigation of interpersonal environment to peer and family environments (Martin et al., 2019). However, materialism develops over an individual’s lifespan (Martin et al., 2019). Individuals face a more complicated and diverse interpersonal environment in adulthood, and it is still possible to form materialistic values. Therefore, the causes of materialism in adulthood need to be discussed. A few studies among college students have suggested that ostracism may be one of the interpersonal environmental factors that lead to materialism in adults (Chen, 2018; Li et al., 2020).

Ostracism refers to rejection or being ignored by others (Williams, 2009). Suffering ostracism will lead to a high level of materialism for several reasons. First, according to the temporal need–threat framework, ostracism makes people feel social pain, increasing their negative affect, and threatening their basic needs (control, belonging, self-esteem, “meaningful existence” needs) (Williams, 2009). Possessions, on the other hand, can alleviate social pain caused by ostracism (Zhou et al., 2009), repair negative emotions (Müller et al., 2012) and meet four basic needs (Zhou and Xie, 2019) in a short time, thus becoming a coping strategy for individuals facing the threat of ostracism. Second, from the resource-gaining perspective, social connections and money are personal resources that can complement each other (Zhou and Gao, 2008). Money activates the sense of self-sufficiency (Vohs et al., 2006), which makes people feel that the problem can be solved without seeking help from others. Therefore, when ostracism cuts off social connections, people may turn to material possessions to compensate for their loneliness, which may further cause materialism. Third, ostracism may cause people to be eager for money (Zhou et al., 2009), and act in a showy way (Chen et al., 2017). Jiang et al. (2015) found that peer rejection increased the level of materialism in children and teenagers. This phenomenon is also typical for young adults, as research on college students showed that those experiencing chronic ostracism had higher scores on materialism (Chen, 2018; Li et al., 2020). Therefore, we propose the following hypothesis:

*Hypothesis 1:* Ostracism positively predicts materialism in adults.

### The Mediation Effect of Security

According to the theory of materialistic values (Kasser et al., 2004), when basic needs cannot be satisfied by circumstances, that insecurity will force people to form materialistic values to compensate for their needs. Security refers to the sense of power to cope with the possible physical or psychological dangers, which exist in both natural environment and social environment (Cong and An, 2004). The premonition of such dangers causes a state of concern with loss (Wichman et al., 2015), and insecurity can arise when individuals feel powerless to cope with such dangers. Security is a general term, which exists in various of contexts, such as attachment security in interpersonal context, existential security in health context, etc., (Wichman et al., 2015). When lacking a sense of security, people may worry about their ability to effectively cope with challenges, and hence pursue material wealth to compensate for it (Kasser et al., 2004; Wang et al., 2019). It is because material wealth can enhance their likelihood of meeting basic needs for safety and sustenance (Kasser et al., 2004), and meeting their needs of seeking confirmation and control (Wang et al., 2016), which can compensate for the sense of security. Empirical research supports the notion that people may have a higher implicit materialistic attitude when they experience existential insecurity (Wang et al., 2016). Attachment insecurity in interpersonal relationships predicts higher materialism (Sun et al., 2020b), while enhancing interpersonal security can reduce an individual’s emphasis on possessions (Clark et al., 2011). Security is an important psychological factor affecting materialism, but previous studies have never explored whether security may be the internal psychological mechanism of ostracism’s influence on materialism. Ostracism, as an environmental factor, attacks individuals’ basic needs, making some people lose their sense of certainty and control over life (Williams, 2009), and become overcautious in interpersonal communication, thus damaging their security (Wesselmann et al., 2015). Güzel and Şahin (2018) concluded that ostracism makes individuals unable to gain recognition from groups to which they belong, and creates uncertainty about themselves, which leads to insecurity. Yaakobi (2018, 2019) further supports this hypothesis, by stating that ostracism brings anxiety and fear of death. In addition, individuals with high rejection sensitivity will have more relational insecurity (Volz and Kerig, 2010). Thus, the present study proposes the following hypothesis:

*Hypothesis 2:* Ostracism predicts materialism through damaging security.

### The Moderation Effect of Self-Construal

The way ostracism affects behavior is not always the same; there may be cultural differences (Uskul and Over, 2017), that are shown as different types of self-construal at the individual level (Markus and Kitayama, 1991). Self-construal relates to the way in which an individual perceives himself or herself to be connected with others in the community (Markus and Kitayama, 1991). Specifically, individualistic culture corresponds to an independent self-construal. People with independent self-construal value that independence, suggesting that the self is isolated from others (Cross et al., 2011). Collectivism culture corresponds to interdependent self-construal. Those with interdependent self-construal value interpersonal relationships and define the self as part of a social network (Cross et al., 2011).

As for the cultural differences of individuals affected by ostracism, the researchers put forward two competitive hypotheses (Uskul and Over, 2017): (A) people with interdependent self-construal are more negatively affected by ostracism, because they value social connections, thus losing them may cause more risks; and (B) people with interdependent self-construal are less negatively affected by ostracism because their social connections can buffer against the pains of ostracism. A few empirical studies support Hypothesis B (Ren et al., 2013; Pfundmair et al., 2015). However, these studies only focused on a single ostracism event in the laboratory, and people with interdependent self-construal are more likely to think of their social bonds to cope with it (Ren et al., 2013; Pfundmair et al., 2015). In the present study, we used the scale to measure the frequency of individuals being ostracized by others. A high score on the scale indicates that an individual has suffered ostracism many times, which will deplete one’s coping resources, cause the individual to withdraw from social interaction and fall into isolation (Williams, 2009). At this time, people with interdependent self-construal would experience more damage because they define themselves through social networks (Cross et al., 2011), and their security comes from social relationships (Kagitcibasi, 1997). Withdrawing from social interaction cuts off the social relationships they value, which in turn threatens their self-concept and basic needs, resulting in a lower sense of security. Therefore, we predict the following:

*Hypothesis 3:* Self-construal moderates the relationship between ostracism and security. More specifically, compared with individuals with independent self-construal, ostracism has a larger impact on security for those with interdependent self-construal.

Meanwhile, self-construal may also moderate the direct relationship between ostracism and materialism. Material possessions can be seen as a way of achieving uniqueness and autonomy (Zhang and Hawk, 2019). Belk (1988) extended-self theory pointed out that the independent self is reinforced through purchasing things, which serve as an extension of an independent and unique identity. Therefore, individuals with independent self-construal originally seek more material possessions than social bonds (Gil et al., 2016) and ostracism may have less influence on materialism. On the contrary, individuals with interdependent self-construal tend to value social bonds more than material possessions (Zhang and Hawk, 2019). For them, only when ostracism makes it difficult to form social bonds, do they turn to material possessions. In other words, ostracism has a greater influence on their materialism. Thus, we propose the following hypothesis:

*Hypothesis 4:* Self-construal moderates the direct relationship between ostracism and materialism. More specifically, compared with individuals with independent self-construal, ostracism has a greater impact on materialism for those with interdependent self-construal.

**FIGURE 1:**
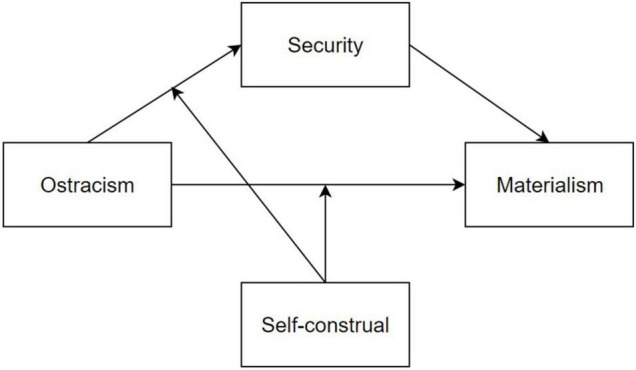
The hypothetical model.

In general, based on temporal need-threat framework (Williams, 2009) and the theory of materialistic values (Kasser et al., 2004), the present study, for the first time, regards security as the mediating factor of the relationship between ostracism and materialism, and further explores the moderating role of self-construal on this process. The hypothesis model is shown in Figure 1.

## Materials and Methods

### Participants and Procedures

The data was collected by college students under unified recruitment. After strict training, the volunteers took questionnaire to their hometown and collected the data.

After screening out individuals who did not complete the survey, or did not pass the lie tests, 1,272 questionnaires remained (47.2% male). The average age was 35.9 (*SD* = 11.59), ranging from 18 to 74. Annual household income was categorized as: below 6,000 Yuan (11.08%), 6,000–15,000 Yuan (8.88%), 15,000–30,000 Yuan (11.56%), 30,000–90,000 Yuan (28.93%), 90,000–150,000 Yuan (20.36%), 150,000–200,000 Yuan (11.40%), 200,000–500,000 Yuan (6.13%), 500,000–1,000,000 Yuan (0.94%), and 1,000,000 Yuan and above (0.71%). Educational qualifications were recorded as: less than a junior high school diploma (18.32%); high school diploma (18.63%); junior college degree (19.42%); bachelor’s degree (37.58%); and master’s degree or higher (6.05%).

### Methods

#### Ostracism

The revised version of the ostracism experience scale (Carter-Sowell, 2010; Shao et al., 2020), which includes eight items, was used to measure chronic ostracism experience. A representative item is “Others leave me out of their groups.” Each item is rated on a seven-point Likert scale ranging from 1 (hardly ever) to 7 (almost always). The Cronbach’s alpha was 0.93.

#### Materialism

The revised version of the materialism value scale (Richins, 2004) was used, with higher scores indicating a higher level of materialism. It includes 15 items (e.g., “Some of the most important achievements in life include acquiring material possessions”). Each item is rated from 1 (strongly agree) to 5 (strongly disagree). The Cronbach’s alpha was 0.67.

#### Security

The security questionnaire (Cong and An, 2004) was used to measure the level of security, with higher scores standing for higher level of security. The questionnaire contains 16 items (e.g., “I never dare to speak out my own opinions”). Each item is rated from 1 (strongly agree) to 5 (strongly disagree). Cronbach’s alpha was 0.88.

#### Self-Construal

The revised version of the self-construal scale (Singelis, 1994; Wang et al., 2008) was used to measure the type of self-construal. It contains two subscales, each of which contains 12 items. The independent self-construal subscale contains items such as “I am willing to be different in many ways.” The interdependent self-construal subscale contains items such as “I often feel that maintaining good interpersonal relationships is more important than my own achievements.” Both subscales are rated from 1 (strongly disagree) to 7 (strongly agree). The Cronbach’s alpha for independent self-construal subscale and interdependent self-construal subscale were 0.76 and 0.86, respectively.

## Results

### Descriptive Statistics

First, we divided different self-construal groups according to their self-construal scores. Participants with a higher independent score were divided into an independent self-construal group (*n* = 296), and those with a higher interdependent score were divided into the interdependent self-construal group (*n* = 880). Participants with equal scores were excluded from further analysis (Liu and Rau, 2014; Yang et al., 2018; Sun et al., 2020a). For the independent self-construal group, the mean of the independent self-construal score is 5.15, and the mean of the interdependent self-construal score is 4.75. For the interdependent self-construal group, the mean of the independent self-construal score is 4.80, and the mean of the interdependent self-construal score is 5.48.

The correlation analysis showed that the main variables were significantly related to each other (see Table 1). Specifically, ostracism was negatively related to security and positively related to self-construal and materialism. Security was negatively related to self-construal and materialism. Self-construal was positively related to materialism.

**TABLE 1:** Descriptive statistics and correlations among all variables.

Variables	*M*	*SD*	1	2	3	4	5	6
1. Sex	–	–	1					
2. Age	35.90	11.59	−	1				
3. Annual household income	4.06	1.73	0.07*	−0.09**	1			
4. Ostracism	2.51	1.11	0.09**	–0.02	−0.17***	1		
5. Security	3.35	0.63	–0.03	0.03	0.04	−0.30***	1	
6. Self-construal	-	-	0.06	−0.08**	0.06*	0.11***	−0.09**	1
7. Materialism	2.93	0.43	0.04	−0.17***	−0.07**	0.22***	−0.22***	0.09**

*Sex: 0 (female), 1 (male); annual household income: 1 (below 6,000 Yuan)–9 (1,000,000 Yuan and above).*

*p < 0.05, **p < 0.01, and ***p < 0.001.

#### Moderated Mediation Analysis

All variables were standardized. Model 4 of the PROCESS macro was used to test the mediation effect of security (Hayes, 2013; Wen and Ye, 2014). After controlling for sex, age, and annual household income, ostracism negatively predicted security (*a* = −0.30, SE = 0.03, *p* < 0.001). Then, ostracism was entered as the predictor, security as the mediator, and materialism as the outcome. The results indicated that ostracism significantly positively predicted materialism (*c*′ = 0.17, SE = 0.03, *p* < 0.001), and security significantly negatively predicted materialism (*b* = −0.16, SE = 0.03, *p* < 0.001). Bootstrap analysis indicated that the mediating effect of security was significant [effect size = 0.05, BootSE = 0.01; 95% CI = (0.03, 0.07)]. The ratio of the mediation effect to the total effect was ab/(ab + c′) = 22.73%.

In addition, we tested the moderating effect of self-construal using Model 8 of the PROCESS macro. The results show that in Model 1 (see Table 2), the total effect of ostracism on materialism was significant. In Model 2, ostracism had a significant effect on security, and the interaction between ostracism and self-construal also had a significant effect on security. Therefore, we concluded that self-construal had a significant moderating effect on the intermediary first half path. In Model 3, ostracism and security had significant effects on materialism, while the interaction between ostracism and self-construal had no significant effect on materialism, so self-construal had no significant moderating effect on the direct path.

**TABLE 2:** The moderated mediation model.

Variables	Model 1 (Y: Materialism)			Model 2 (M: Security)			Model 3 (Y: Materialism)		
	β	*SE*	*t*	β	*SE*	*t*	β	*SE*	*t*
Sex	0.02	0.06	0.88	0.03	0.05	0.99	0.02	0.06	0.79
Age	–0.17	0.00	−6.19***	0.00	0.00	0.15	–0.16	0.00	−5.67***
Annual household income	–0.04	0.02	–1.42	0.02	0.02	0.63	–0.05	0.02	–1.77
Ostracism	0.22	0.03	7.79***	–0.35	0.03	−10.50***	0.15	0.04	4.28***
Security							–0.16	0.03	−5.42***
Self-construal				–0.07	0.06	−2.39*	0.05	0.07	1.85
Ostracism × Self-construal				0.09	0.06	2.71**	–0.01	0.07	–0.34
*R* ^2^	0.08			0.11			0.10		
*F*	27.34***			22.81***			18.43***		

*p < 0.05, **p < 0.01, and ***p < 0.001.

The moderated mediation effect was significant [index = −0.03, Boot*SE* = 0.01, 95% CI = (−0.06, −0.01)]. For people with interdependent self-construal, the mediating effect of security was significant [index = 0.06, Boot*SE* = 0.01, 95% CI = (0.04, 0.09)]. For those with independent self-construal, the mediating effect of security was weaker, but also significant [index = 0.03, Boot*SE* = 0.01, 95% CI = (0.01, 0.06)].

**FIGURE 2:**
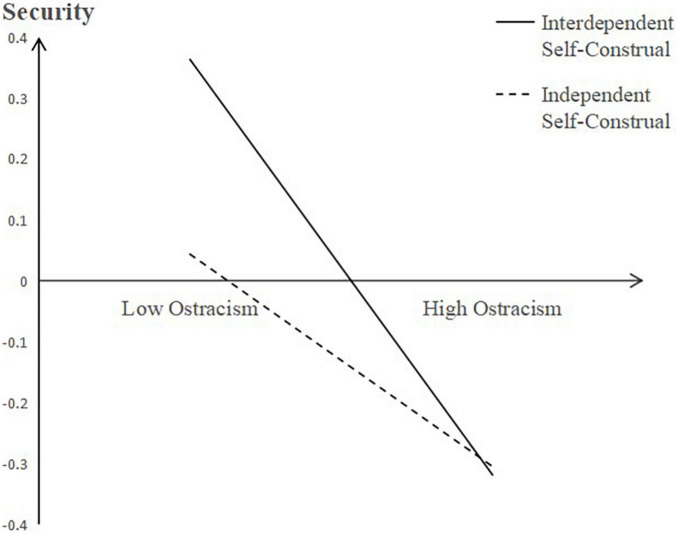
Self-construal as a moderator in the relationship between ostracism and security.

Furthermore, we ran a simple slope test to reveal the moderating effect of self-construal on the relationship between ostracism and security (see Figure 2). The results showed that for people with interdependent self-construal, ostracism had a significant negative effect on security (*B* = −0.35, *t* = −10.50, *p* < 0.001). For those with independent self-construal, the negative effect was weaker, but also significant (*B* = −0.18, *t* = −3.30, *p* < 0.01).

## Discussion

Consistent with previous studies (Zhou et al., 2009; Chen, 2018), ostracism significantly positively predicted materialism. Social support and material possessions are personal resources that can repair and supplement each other (Zhou and Gao, 2008). When ostracism makes people unable to acquire support from others, they pursue material possessions. However, if we see the outcome in the long term, money and social support may have different effects. Chasing after material possessions is endless, damaging long-term happiness (Muñiz-Velázquez et al., 2017), causing one to fall into a vicious circle of pain. Furthermore, the result that ostracism increases materialism can explain why ostracism reduces prosocial behaviors and increases aggressive behaviors. Ostracism may overrate material possession so that its victims donate less for others (Twenge et al., 2007), and more aggressively pursue their own profits (Jiang and Chen, 2020).

It is notable that security mediates the relationship between ostracism and materialism. Ostracism, as an environmental factor, causes higher materialism by reducing security. The result confirms the theory of materialistic values (Kasser et al., 2004), and innovatively integrates it into the temporal needs-threat framework (Williams, 2009). Chronic exposure to ostracism makes people isolate themselves from social networks, leading to a sense of alienation, worthlessness, depression, and learned helplessness (Riva et al., 2017), falling into long-term insecurity (Wesselmann et al., 2015). To compensate for this insecurity, individuals will pay more attention to material wealth that can guarantee their own needs, thus showing higher materialistic values. In addition, there may be other mediating variables between ostracism and materialism, such as relative deprivation, a perception that individuals percept themselves as disadvantaged comparing to others (Smith and Huo, 2014). Ostracism can increase relative deprivation (Jiang and Chen, 2020), which leads to increased materialism (Zhang et al., 2015).

Self-construal acted as a moderator between ostracism and security. Specifically, compared with people with independent self-construal, ostracism had more negative effects on those with interdependent self-construal. Individuals with interdependent self-construal define themselves through social networks and have a stronger need for belonging and relationships (Cross et al., 2011). Ostracism makes them lose their frame of reference to confirm their self-concept and value, and deprives them of the source to meet their psychological needs, thus resulting in greater insecurity. In addition, the present study makes some theoretical contributions. The theory of materialistic values (Kasser et al., 2004) only emphasizes the effect of the environment on security, without considering individual differences in the process. This study goes a step ahead, assuming that individuals interact with the environment (Graziano et al., 2007), and confirming that the effect of the environment (i.e., ostracism) on security is moderated by self-construal. However, the moderating effect of self-construal on the relationship between ostracism and materialism was not significant. A possible reason is that, although ostracism poses a greater threat to people with interdependent self-construal, they may adopt social strategies such as rebuilding interpersonal relationships to deal with the threat (Over and Uskul, 2016) without necessarily increasing their pursuit of material possessions (Zhou and Gao, 2008). Therefore, self-construal did not play a moderating role in the direct impact of ostracism on materialism.

This study has several important implications. First, based on Williams’s temporal needs–threat model (Williams, 2009) and the theory of materialistic values (Kasser et al., 2004), this study, for the first time, investigated the psychological mechanism between ostracism and materialism from the perspective of security, and further discovered the moderating effect of self-construal in this process. By completely explaining the complex mechanism between ostracism and materialism, this study extends and deepens the understanding of the relationship between ostracism and materialism. Second, by focusing on a sample of Chinese adults, this study expanded the age range and population composition of existing research samples. Third, from a practical perspective, the findings provide a reference to reduce and prevent materialism among residents. Specifically, a harmonious social environmental atmosphere, less ostracism, and a higher sense of control and security may prevent materialism. According to the moderating effect of self-construal, guiding individuals not to rely entirely on their relationships with others, and ensuring one’s security through improving self-competence may alleviate the damage to security by ostracism, further reducing the impact on materialism.

### Limitations and Future Research

This study has several limitations. First, the participants with independent self-construal were limited, which may be related to the Chinese background of the study (Hu et al., 2012). Future research could investigate the moderating role of self-construal in intercultural backgrounds. Second, self-rated questionnaires were used to measure all variables, including negative features, and, therefore, the results may have been influenced by social desirability results. To address this concern, future studies can combine various evaluation methods, such as peer evaluation. Third, as a cross-sectional study, the causal effect could not be confirmed. The relationship between ostracism and materialism may also be bidirectional. Specifically, materialists are considered to be more selfish and self-centered (Shrum et al., 2014), which may hinder the achievement of group goals, leading to being ostracized by group members (Rudert et al., 2020). Thus, an experimental design and longitudinal studies are needed.

## Data Availability Statement

The raw data supporting the conclusions of this article will be made available by the authors, without undue reservation.

## Ethics Statement

The studies involving human participants were reviewed and approved by the Ethics Committee in the Department of Psychology, Renmin University of China. The patients/participants provided their written informed consent to participate in this study. Written informed consent was obtained from the individual(s) for the publication of any potentially identifiable images or data included in this article.

## Author Contributions

JF, YW, and DZ conceived and designed the study. DZ and ZJ collected the data and revised the manuscript. JF analyzed the data. JF and YW contributed to the writing of the manuscript. All authors contributed to the article and approved the submitted version.

## Conflict of Interest

The authors declare that the research was conducted in the absence of any commercial or financial relationships that could be construed as a potential conflict of interest.

## Publisher’s Note

All claims expressed in this article are solely those of the authors and do not necessarily represent those of their affiliated organizations, or those of the publisher, the editors and the reviewers. Any product that may be evaluated in this article, or claim that may be made by its manufacturer, is not guaranteed or endorsed by the publisher.
