# Supplementary material for: The Effect of Ostracism on Adults’ Materialism: The Roles of Security and Self-Construal
Source: Front Psychol. 2022 Apr 18;13:796924. doi: 10.3389/fpsyg.2022.796924 (PMC9062183; doi:10.3389/fpsyg.2022.796924)
Supplement: Supplementary file 1 [file Data_Sheet_1.docx]

**APPENDIX**

**指导语：**

**Guidance:**

您好！我们正在进行一项社会调查，目的是了解人们对当前社会生活状况的真实评价和感受，您的合作对于我们了解社会现状和相关政策的制定有着十分重要的意义。对于您的回答，我们将按照《统计法》的相关规定严格保密，并且只用于统计分析，请您不要有任何顾虑。问卷中所有问题的回答没有对错之分，您只需要根据您的第一反应给出答案，因此请您仔细阅读每一个题目，在合适的答案上画“√”。请不要遗漏任何一道题。感谢您的支持和帮助！

Hello! We are conducting a social survey to find out people's true evaluation and feelings about the current social living conditions. Your cooperation is of great significance for us to understand the current social situation and formulate relevant policies. Your answers will be kept strictly confidential in accordance with the relevant provisions of the Statistics Law, and will only be used for statistical analysis. Please do not have any concerns. There is no right or wrong answer to all questions in the questionnaire. You only need to give the answer according to your first reaction, so please read each question carefully and draw “√” on the appropriate answer. Please don't miss any questions. Thank you for your support and help!

**社会排斥体验量表**

**Ostracism Experience Scale**

1. 别人总是将我排除在外。

Others leave me out of their groups.

1. 别人常常对我封锁很重要的信息。

Others keep me out-of-the-loop on information that is important to my close relationships.

1. 别人好像经常看不到我似的。

Others treat me as if I were invisible.

1. 别人总是对我很冷淡。

Others give me the cold shoulder treatment.

1. 当我出现时，别人常常会背过身去。

Others physically turn their backs to me when in my presence.

1. 别人对待我的态度，经常像我被单独关了禁闭一样。

Others treat me as if I’m in solitary confinement.

1. 当我出现时，别人基本都不会看我。

Others do not look at me when I’m in their presence.

1. 在大家一起交谈时，我常常被别人忽视。

Others ignore me during conversation.

**物质主义价值观量表**

**Materialism Value Scale**

1. 我佩服那些拥有昂贵房屋、汽车和衣服的人们。

I admire people who own expensive homes, cars, and clothes.

1. 我拥有了享受生命所需的所有东西。

I have all the things I really need to enjoy life.

1. 获得物质财产是生命中最重要的成就之一。

Some of the most important achievements in life include acquiring material possessions.

1. 我努力让自己生活中拥有的物品尽可能保持简单。

I try to keep my life simple, as far as possessions are concerned.

1. 如果拥有某些现在没有的东西 , 我的生活会更好。

My life would be better if I owned certain things I don’t have.

1. 我并不强调将人们拥有的物质财富的数量作为他们成功的标志。

I don’t place much emphasis on the amount of material objects people own as a sign of success.

1. 假使我拥有更好的东西 , 我并不会因此而更加快乐。

I wouldn’t be any happier if I owned nicer things.

1. 在很大程度上, 我拥有的东西可以表明我在生活中表现如何。

The things I own say a lot about how well I’m doing in life.

1. 我享受花钱购买不实用的东西。

I enjoy spending money on things that aren’t practical.

1. 如果我能买得起更多的东西 , 我会更加快乐。

I’d be happier if I could afford to buy more things.

1. 我喜欢拥有那些给别人以深刻印象的东西。

The things I own say a lot about how well I’m doing in life.

1. 购物给我带来很多乐趣。

Buying things gives me a lot of pleasure.

1. 我买不起所有想要的东西 , 我时常为此感到烦恼。

It sometimes bothers me quite a bit that I can’t afford to buy all the things I’d like.

1. 我喜欢自己的生活中有很多奢侈品。

It sometimes bothers me quite a bit that I can’t afford to buy all the things I’d like.

1. 比起我认识的很多人来说 , 我不那么重视物质的东西。

I put less emphasis on material things than most people I know.

**安全感量表**

**Security Questionnaire**

1. 我从来不敢主动说出自己的看法。

I never dare to speak out my own opinions.

1. 我感到生活总是充满不确定性和不可预测性。

I feel that life is always full of uncertainty and unpredictability.

1. 我习惯于放弃自己的愿望和要求。

I am used to giving up my wishes and demands.

1. 我总是会担心发生什么不测。

I always worry about what will happen.

1. 我从不敢拒绝朋友的要求。

I never dare to refuse a friend's request.

1. 遇到不开心的事，我总是独自生闷气或痛哭。

When I encounter unhappy things, I always sulk or cry alone.

1. 我一直觉得自己挺倒霉的。

I've always been feeling that I was unlucky.

1. 人们说我是一个害羞、退缩的人。

People say that I am a shy and isolated person.

1. 我总是担心太好的朋友关系以后会变坏。

I always worry if a friend relationship is too good, it would go bad in the future.

1. 对领导我一般是敬而远之。

I usually stay at a respectful distance from leaders.

1. 我常常担心自己的思维或情感会失去控制。

I often worry that my thoughts or emotions will get out of control.

1. 我总是“万事不求人”。

I do not ask for help about anything.

1. 我总是担心自己的生活会变得一团糟。

I always worry that my life will become a mess.

1. 我感到自己无力应对和处理生活中突如其来的危险。

I feel that I am unable to cope and deal with the unexpected dangers in my life.

1. 我害怕与他人建立并保持亲近关系。

I'm afraid to establish and keep close relationship with others.

1. 无论别人怎么说，我都觉得自己很没用。

No matter what others say, I feel useless.

**自我建构量表**

**Self-construal Scale**

1. 我敬重我所交往的权威人物。

I admire the authorities I have met.

1. 对我来说，与他人维持一种融洽的关系非常重要。

It is very important for me to maintain a harmonious relationship with others.

1. 周围人的快乐就是我的快乐。

The happiness of people around you makes me happy.

1. 为了集体的利益，我会牺牲自己的利益。

I will sacrifice my own interests for the benefit of the collective

1. 我经常感到保持良好的人际关系比我自己取得的成绩更重要。

I often feel that maintaining good interpersonal relationships is more important than my own achievements.

1. 对我来说，尊重集体的决定是重要的。

It is important for me to respect the collective decision.

1. 当众发言对我来说不成问题。

Speaking in front of the public is not a problem for me.

1. 对我来说，保持活跃的想象很重要。

It is very important for me to keep an active imagination.

1. 我在家里和在单位里的表现始终如一。

My performance at home and at work is consistent

1. 与刚认识的人交往时，我喜欢直截了当。

I like to be direct when dealing with people I just met.

1. 我乐意在许多方面与众不同。

I am willing to be different in many ways.

1. 独立于他人的个性特点对我来说是非常重要的。

Individuality is very important to me.

1. 乘车时我会主动给老人让座。

I will take the initiative to give my seat to the elderly when riding.

1. 我敬重那些谦虚的人。

I respect those who are modest.

1. 当制定重要计划时我应该考虑家人的建议。

I should consider my family's suggestions when making important plans.

1. 如果我所在的群体需要我，即使我呆得不开心，我也会留在那里。

If my group needs me, I will stay there even if I am unhappy

1. 如果朋友遇到挫折，我觉得我有责任帮助他（她）。

If a friend encounters setbacks, I feel it my duty to help him or her

1. 即使我的观点与群体成员不一致，我也会避免争论。

Even if my views do not agree with the group members, I will avoid arguing.

1. 当我被单独表扬或奖励时，我感到舒服。

I feel comfortable when I am praised or rewarded alone.

1. 与其被误解，不如直截了当地说出自己的想法。

Rather than being misunderstood, it's better to say what you think directly.

1. 对我来说，我主要关心的是能够照顾我自己。

For me, my main concern is to be able to take care of myself.

1. 不管和谁在一起我的表现始终如一。

No matter who I am with, my performance is consistent.

1. 当见到相识不久的人时，我就自然地直呼其名，即使他们的年龄比我大得多。

When I meet with someone I haven't met for a long time, I naturally call them by their first names, even though they are much older than me.

1. 我认为健康是最重要的。

I think health is the most important.
